# Reversion of the ELISPOT test after treatment in Gambian tuberculosis cases

**DOI:** 10.1186/1471-2334-6-66

**Published:** 2006-03-30

**Authors:** Alexander M Aiken, Philip C Hill, Annette Fox, Keith PWJ McAdam, Dolly Jackson-Sillah, Moses D Lugos, Simon A Donkor, Richard A Adegbola, Roger H Brookes

**Affiliations:** 1 Tuberculosis Division, Medical Research Council Laboratories, Banjul, The Gambia

## Abstract

**Background:**

New tools are required to improve tuberculosis (TB) diagnosis and treatment, including enhanced ability to compare new treatment strategies. The ELISPOT assay uses *Mycobacterium tuberculosis-*specific antigens to produce a precise quantitative readout of the immune response to pathogen. We hypothesized that TB patients in The Gambia would have reduced ELISPOT counts after successful treatment.

**Methods:**

We recruited Gambian adults with sputum smear and culture positive tuberculosis for ELISPOT assay and HIV test, and followed them up one year later to repeat testing and document treatment outcome. We used ESAT-6, CFP-10 and Purified Protein Derivative (PPD) as stimulatory antigens. We confirmed the reliability of our assay in 23 volunteers through 2 tests one week apart, comparing within and between subject variation.

**Results:**

We performed an ELISPOT test at diagnosis and 12 months later in 89 patients. At recruitment, 70/85 HIV-negative patients (82%) were ESAT-6 or CFP-10 (EC) ELISPOT positive, 77 (90%) were PPD ELISPOT positive. Eighty-two cases (96%) successfully completed treatment: 44 (55%; p < 0.001) were EC ELISPOT negative at 12 months, 17 (21%; p = 0.051) were PPD ELISPOT negative. Sixty (73%) cured cases had a CFP-10 ELISPOT count decrease, 64 (78%) had an ESAT-6 ELISPOT count decrease, 58 (70%) had a PPD ELISPOT count decrease. There was a mean decline of 25, 44 and 47 SFU/2 × 10^5 ^cells for CFP-10, ESAT-6 and PPD respectively (p < 0.001 for all). Three of 4 HIV positive patients were cured, all 3 underwent ELISPOT reversion; all 4 not cured subjects (3 HIV-negative, 1 HIV positive) were ESAT-6, CFP-10 and PPD ELISPOT positive at 12 months.

**Conclusion:**

Successful tuberculosis treatment is accompanied by a significant reduction in the *M. tuberculosis*-specific antigen ELISPOT count. The ELISPOT has potential as a proxy measure of TB treatment outcome. Further investigation into the decay kinetics of T-cells with treatment is warranted.

## Background

Tuberculosis (TB) causes an estimated 2 million deaths per year [1], the overwhelming majority occur in developing countries [2], and *Mycobacterium tuberculosis *infects approximately one third of the world's population [3]. While the incidence of TB has stabilised or is on the decrease in many parts of the world, the incidence rate is rising by approximately 6% per year in Africa [4]. New diagnostic tools for TB, new and enhanced treatment strategies, plus efficacy markers to compare them, are needed to help combat the epidemic. These need to be shown to be useful in TB-endemic tropical settings.

T-cell based immuno-assays have promise for the diagnosis of TB disease and *M. tuberculosis *infection. The *ex-vivo *ELISPOT can precisely enumerate interferon-gamma (IFN-γ) producing T-cells sensitised to *M. tuberculosis *antigens and we have shown that the quantitative ELISPOT count reflects the infectious load of *M. tuberculosis *in The Gambia [5]. Noting this property, we hypothesized that the ELISPOT count would decrease as a response to a course of anti-TB therapy in TB cases. Therefore, as a first step towards identifying whether the ELISPOT might be an efficacy marker in this way in The Gambia, we assessed the ELISPOT response in TB cases before and after treatment.

## Methods

### Participants

Nested in a large case contact study in The Gambia [6], we recruited sputum smear and culture positive adult TB cases for the study. We took a 10 ml blood sample from each case at the time of diagnosis and followed them up 12 months later to take a repeat sample. The National TB Control Program gave each case a standard six-month course of free treatment by Directly Observed Therapy, short course (DOTS) to all patients. Ethical approval for this study was obtained from the joint MRC – Gambian Government Ethics board. Informed consent was obtained from all study participants.

We tracked the patients' clinical outcome through follow-up interviews and corroborated the results with clinical records (TB treatment cards and clinic treatment register). Patients who were found at follow-up to have successfully completed their treatment, and who were well at time of 12-month blood test (no cough/sweats/weight loss, or other TB symptoms since the completion of treatment), were categorised as "cured." Others were further categorised according to standard WHO definitions [7]: they were defined as "defaulted" if treatment was interrupted for two consecutive months or they were defined as "failed treatment" if they remained sputum smear positive at 4 months or later during a course of treatment.

### Laboratory procedures

Sputum smears were prepared and stained with auramine-phenol [8] and the results were confirmed by Ziehl-Neelsen staining. Decontaminated specimens were inoculated into Lowenstein-Jensen medium and BACTEC 9000 liquid medium for the isolation and identification of M. tuberculosis, as described previously [9]. HIV testing was done by enzyme linked immunosorbent assay (ELISA; Wellcome Laboratories, Dartford, Kent, UK) and by type-specific Western blot (New LAV Blot I and New LAV Blot II, Diagnostics Pasteur, Marnes-la-Coquette, France). All data were entered using double data entry into an ACCESS database and verified.

The ex-vivo ELISPOTs were performed in duplicate as previously described [10]. Overlapping peptides spanning the length of ESAT-6 and CFP-10 (ABC, Imperial College, London, UK) were each divided into peptide pools, and used at 5 μg/ml. Purified Protein Derivative (PPD; *Mtb*, RT49, Statins Serum Institut, Copenhagen, Denmark) was used at 10 μg/ml. The positive control was Phytohaemaglutinin (PHA; Sigma-Aldrich, UK). Assays were scored by an automated ELISPOT reader (AID-GmbH, Strassberg, Germany). A positive test well was pre-defined as containing at least 10 Spot Forming Units (SFUs) more than, and at least twice as many as, negative control wells. PHA wells were set to at least 150 SFUs above negative control wells, which were required to have less than 30 SFUs to be included in the analysis. Antigen SFUs were reported as number of SFUs above the negative control SFUs.

### Reliability of the ELISPOT assay

We assessed the reliability of the ELISPOT assay in two ways. Firstly, to assess the within-subject variability, we tested each antigen in duplicate for each subject and compared the within and the between subject variability by fitting a random effects model for subject. Within our large case contact study, we used ELISPOT results from consecutively recruited TB case contacts: 1223 duplicate results for ESAT-6 peptides and 1182 duplicate results for CFP-10 peptides. For CFP-10 the between subject standard deviation was 29.4 spots and the within subject standard deviation was 3.6 spots. For ESAT-6, the between-subject standard deviation was 38.9 spots and the within-subject standard deviation was 4.2 spots. Therefore the within-subject variability was very small compared to the between-subject variability. Secondly, to assess the day-to-day variability, we recruited 23 adult volunteers from the general community and conducted an ELISPOT test followed by a repeat test after 1 week. Twelve volunteers had a negative test initially: none of these had a positive test after 1 week. Eleven volunteers had a positive test initially: only one of these had a negative test after one week. For those who were positive at both time points the differences between the SFUs at the two time points were: -15, -7, -4, -1, 3, 5, 6, 6, 12, and 35 SFU respectively.

### Data management and analysis

All data were entered via double data entry into an Access database and checked for errors. Statistical analysis was conducted using SPSS software. Qualitative outcomes were compared by a test of proportions and quantitative outcomes by student's t-test (paired). When necessary (for ESAT-6 and CFP-10) log (base 10) transformations were performed.

## Results

We identified 151 sputum smear and culture positive subjects who had a satisfactory ELISPOT test result from blood tests taken at the time of diagnosis. At 12 months, 97 (64%) of these cases were found, agreed to be bled and had a blood sample taken. From these, a satisfactory ELISPOT result was obtained in 89 subjects (92% technical success); 7 had a failed ELISPOT test because of a weak positive control count and one had too high a negative control count. Table 1 shows the basic characteristics of the 89 cases included in the study. Sixty-two (70%) were men and the mean age was 32 yrs (range 18 – 80 yrs). Four cases (4%) were HIV positive.

**Table 1:** Demographics of 89 smear and culture positive tuberculosis patients

Characteristic	(n = 89)
All patients	
Age, years	
Mean	32
Median (range)	26 (18 – 80)
	
Male Sex	62 (70%)
	
Ethnic group	
Mandinka	40 (45%)
Jola	15 (17%)
Wolof	13 (15%)
Fula	11 (12%)
Other	10 (11%)
	
HIV-negative	85/89
Outcome of TB treatment	
Completed treatment and cured	82/85 (96%)
Defaulted from treatment	2/85 (3%)
Treatment failed (remained sputum +ve)	1/85 (1%)
	
HIV-positive	4/89
Outcome of TB treatment	
Completed treatment and cured	3/4 (75%)
Defaulted from treatment	1/4 (25%)

**Figure 1:**
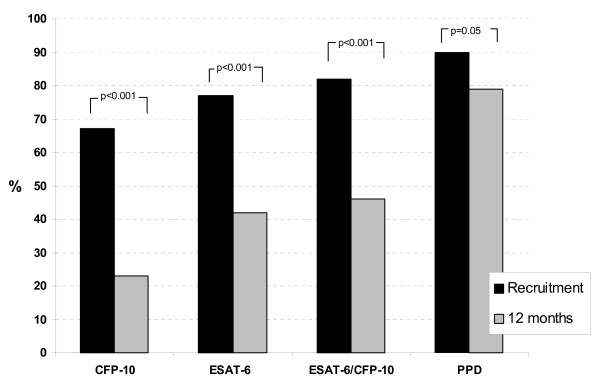
The proportions of cured sputum positive TB cases that have positive ESAT-6, CFP-10 or PPD ELISPOT test at recruitment (diagnosis) and 12 months later (n = 82). P values shown in figure are from tests of proportions.

Figure 1 shows the qualitative ELISPOT results at recruitment and 12 months in the 85 HIV negative cases. At recruitment, 70 cases (82%) were ESAT-6 or CFP-10 (EC) ELISPOT positive, and 77 (90%) were PPD ELISPOT positive. Eighty-two subjects (96%) successfully completed the 6-month TB drug-treatment regimen and were considered to be cured. Of these, 44 (54%) were EC ELISPOT negative at 12 months, and 17 (21%) were PPD ELISPOT negative. For ESAT-6 and CFP-10, the differences between recruitment and 12 months were highly significant (p < 0.001, test of proportions). The difference in PPD ELISPOT positivity was of borderline significance (p = 0.051). Three cured cases were EC ELISPOT negative at recruitment but positive at 12 months whereas 7 cured cases were PPD ELISPOT negative at recruitment but positive at 12 months. There were no significant differences in age, sex or ethnicity between the 34 cured cases who had a positive EC ELISPOT test at both recruitment and at 12 months and those who either reverted from positive to a negative ELISPOT, or were negative at both time points (data not shown).

**Figure 2:**
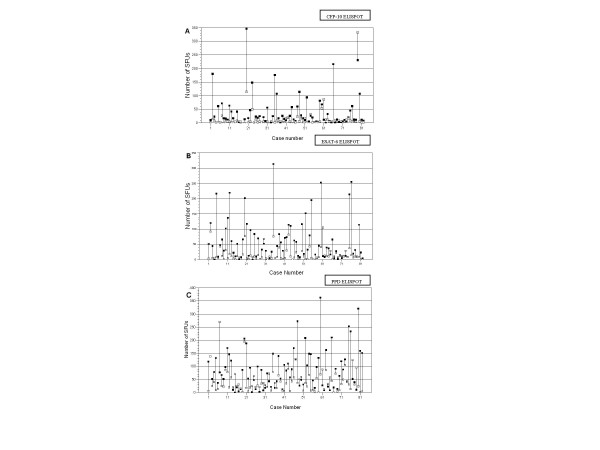
Paired recruitment and 12 months quantitative counts of Spot Forming Units (SFU)/2 × 10^5 ^cells in ELISPOT tests using CFP-10 (Fig. 2a), E-SAT6 (Fig. 2b) and PPD (Fig. 2c) as stimulatory antigens for cured, HIV-negative patients (n = 82). Filled black squares (■) represent the SFU count at recruitment; white squares (□) represent SFU counts 12 months later, after successful completion of TB treatment. The change in counts between the two timepoints is represented by the linking vertical bar. With all three stimulatory antigens, the average within-subject decline in SFU counts is highly significantly (p < 0.001).

Figure 2 shows the quantitative change in the ELISPOT counts between recruitment and 12 months for the 82 cured cases. Sixty (73%) cases had a decrease in their CFP-10 ELISPOT count at 12 months and 64 (78%) had a decrease in their ESAT-6 ELISPOT count. Fifty-eight (70%) cured cases had decrease in their PPD ELISPOT counts. For the three tests, there was a mean decline in counts at 12 months of 25, 44 and 47 SFU/2 × 10^5 ^cells for CFP-10, ESAT-6 and PPD respectively, all highly significant (p < 0.001). Six (7%) cured cases had an increase in either their ESAT-6 or CFP-10 ELISPOT count of at least 10 SFU (Figure 2a and 2b), including one who had an increased count for both antigens; 18 (21%) cases, had an increased PPD ELISPOT count of at least 10 SFU (Figure 2c) which included 4 of the 6 with increased EC counts.

Three HIV-negative cases were defined as "not cured". Two were defined as "defaulted from drug therapy"; both subsequent died with symptoms suggestive of TB recurrence after the 12-month blood test. One case was defined as "treatment failure": he was sputum-smear positive at the end of his six months of TB treatment despite good compliance. All three of these subjects were ESAT-6, CFP-10 and PPD ELISPOT positive at 12 months.

Three of four HIV positive cases completed the TB treatment course and were healthy at the time of the 12-month testing. The EC ELISPOT tests in all 3 reverted from positive to negative. One HIV-positive subject defaulted from TB therapy: all three of his ELISPOT tests (CFP-10, ESAT-6 and PPD) were positive at 12 months.

## Discussion

As far as we are aware this is the largest study so far to evaluate ELISPOT reversion after anti-TB treatment. A significant proportion of patients who successfully completed a course of standard TB treatment changed from a positive to a negative EC ELISPOT 12 months after their initial diagnosis. There was also a significant reduction in mean ESAT-6, CFP-10 and PPD ELISPOT counts. Seven per cent of cases had an increase in their ELISPOT counts for either ESAT-6 or CFP-10, compared to 21% for PPD.

The data reported here cannot be explained by the inherent variability of the ELISPOT readout and are consistent with those from other, albeit small, studies from other settings. Two previous studies of tuberculosis patients in the United Kingdom (UK; n = 4) and Italy (n = 18) reported a decline in quantitative ESAT-6 ELISPOT counts after drug treatment [11,12], However, in one UK study, ESAT-6 ELISPOT counts in 10 patients who had completed TB therapy were marginally but not significantly higher than the counts in 8 different patients early in their TB treatment course [13].

The *ex vivo *ELISPOT response is thought to identify recently activated lymphocytes with immediate effector memory that persist only a limited time in circulation once antigen is cleared [14]. It follows therefore that antigen withdrawal after anti-TB therapy would be accompanied by a decline in T cell frequencies. There are several possible explanations why even 'specific' antigen (ESAT-6 and CFP-10) counts did not universally drop below the pre-defined, and relatively conservative, cut-off for positivity (10 SFU) in The Gambia. Firstly, some patients may have been re-exposed to *M. tuberculosis *after the completion of treatment and re-aquired latent *M. tuberculosis *infection. Secondly, they may have been exposed to environmental mycobacteria, commonly encountered in the tropics, which share the esat-6 and cfp-10 genes (eg. *M. marinum*). Thirdly, and most intriguingly, there is evidence that in some individuals there is persistence of a population of activated T cells in the absence of direct mycobacterial antigen stimulation, even for several years after completing therapy [15].

Having determined that, even in this TB-endemic tropical setting, ELISPOT counts decrease significantly in TB cases after treatment, we will explore the decay kinetics of specific antigen T-cell responses during the course of treatment. It has been suggested that the decay rate of antigen-specific T-cells is approximately 5.5% of the total count per week, [11] which would lead to an approximately 90% decline in counts over 40 weeks. In a small study in South Africa, Nicol et al showed that 10 children with TB had an initial rise, then a decline in ELISPOT counts over a 6-month treatment course [16]. Vekemans et al, in The Gambia, showed that TB patients had relatively low T cell responses to purified mycobacterial antigens, compared to healthy controls with evidence of *M. tuberculosis *infection [17]. An obvious explanation for these findings is that patients at diagnosis have suppressed T cell responses because they are unwell. *M. tuberculosis *can itself interfere with IFN-γ signalling [18], providing another possible explanation. Other factors may also be important, such as increased early antigen presentation as a result of pathogen killing at the onset of antibiotic therapy. Thus the implied trajectory of cellular kinetics is of T-cell responses that increase early in treatment, then decline as the *M. tuberculosis *antigen load diminishes. A possible sampling frame would be to conduct repeat ELISPOT tests at 1 month, 2 months (to compare with sputum conversion in smear positive cases), 4 months and 6 months. We also aim to follow those who are persistently ELISPOT positive as a cohort over time.

While it is of interest that all four of the treatment failures (3 HIV-negative, 1 HIV-positive) in our study had positive EC and PPD ELISPOT tests at 12 months, larger numbers of similar cases will be required to identify the true sensitivity of ELISPOT persistence with respect to treatment failure. That a few cured cases had a rise in ELISPOT counts for ESAT-6 and/or CFP-10 suggests that the positive predictive value of this finding for treatment failure is not 100% in The Gambia. However, it is quite possible that 'cured patients' may relapse at a later date.

## Conclusion

There is now compelling evidence from a variety of settings that a significant decrease in the ELISPOT count occurs in TB patients that are cured by TB treatment. This may have important implications for clinical practice: effective monitoring of response to treatment, tailoring the duration of anti-tubercular drug therapy, comparing the efficacy of new therapies (including therapeutic vaccines), and rationalising the basis for treatment of latent infection are all possible applications. Further studies are required to define the sensitivity and predictive value of ELISPOT reversion and persistence in relation to TB treatment, to find the best time to conduct a repeat test, and to identify clinical and immunological risk factors for persistence of an ELISPOT response and whether such individuals are at any risk of disease recurrence. A repeat ELISPOT test at 12 months reflects the efficacy of a course of anti-TB treatment even in a TB endemic tropical setting.

## Competing interests

RHB has a patent relating to *ex vivo *ELISPOT licensed through Oxford University. There is no other known conflict of interest.

## Authors' contributions

AA supervised the follow-up visits, drafted the manuscript and conducted the analysis. PH was involved in the design of the study, supervised the fieldwork and drafted the manuscript with AA. AF was involved in the design of the study and co-supervised the laboratory work. KM was involved in the design of the study and participated in the coordination of both the field and laboratory aspects. DS supervised the field work and ran the clinical aspects of the study. ML conducted the immunological assays. SD managed the data entry and verification and quality. RA supervised the microbiological aspects of the study. RB was involved in the design of the study and supervised the laboratory aspects. All authors read and approved the final manuscript.

## Pre-publication history

The pre-publication history for this paper can be accessed here:


